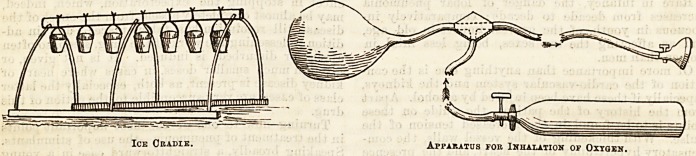# The Treatment of Pneumonia

**Published:** 1893-11-18

**Authors:** 


### THE HOSPITAL CLINIC.

*[The Editor will be glad to receive offers of co-operation and contributions from members of the profession. All letters should be addressed to* THE EDITOR, THE LODGE, PORCHESTER SQUARE, LONDON, W.]

### LONDON HOSPITAL.

### THE TREATMENT OF PNEUMONIA.

In the present day pneumonia is looked upon as a definite form of fever, the pathological manifestations of which are to be found chiefly in the lungs.

Any attempt to shorten the course of the disease is now recognised to be futile; our aim is to guide the patient through the course of a definite fever, placing him under such circumstances as will enable him to withstand the attack with the least drain on his tissues; to watch, and by judiciously timed treatment prevent the failure of such of the vital organs as are tending to succumb. This, though summed up in a few words, embraces in reality wide and often widely differing plans of treatment, varied according to the condition of the patient and the individual predilections of different physicians.

Prognosis and treatment are intimately associated in pneumonia, the same points that tell us how the case will progress on the one hand, giving indications for lines of treatment on the other; so it will be well to glance for a moment at some points to be watched during the course of the disease, as by these points our treatment will be influenced.

Rare in infancy, the danger of lobar pneumonia increases from decade to decade; comparatively innocuous in youth, of the gravest import in old age, and as affecting the two sexes, being less fatal in women than men.

Of more importance than anything else is the condition of the cardio-vascular system and the kidneys, especially if these have been injured by alcohol. Apart from the history of the patient, the guide on these points is to be found in the increased tension of the pulse. With thickening of the vessel walls, the compensatory hypertrophy of the heart, and the presence of albumen and casts in the urine—a trace of albumen in the urine during the fever, is part of the natural course of the disease—but the persistent presence of albumen with casts, and a urine of uniformly low specific gravity points to serious organic change and a much graver prognosis.

Serious heart disease of any description, or chronic disease of the lungs, are, of course, points that will influence us unfavourably in prognosis, and modify somewhat our treatment. Passing now to the details of treatment: of primary importance is rest in bed, not merely because the patient may feel too ill to be about, but because both heart and lungs, the two organs upon which fall the stress of the disease, have least work to do when the patient is in the recumbent position, and the nervous system—regulator of the action of these organs, has less call made upon its reserve of energy. Care is taken to keep as equable a temperature in the ward as possible, with the avoidance of draughts.

With these precautions and suitable nourishment, an ordinary case of pneumonia in a healthy young subject will progress to convalescence with little, if any, drug treatment. An aperient is usually given on admission, or at the beginning of the disease, and for this purpose calomel or Epsom salts are most frequently used; it ensures the emptying of the intestinal tract, and places the stomach, whose powers of assimilation are already somewhat impaired by the high temperature of the body, in the best position to digest the necessary nourishment. As a rule, a certain amount of pleurisy is associated with the pneumonia, and causes pain on breathing, most often felt as a stitch or stabbing pain a little outside and below the nipple, or else about the lower angle of the scapula behind; this pain is often very severe, and its relief becomes imperative. Various plans are used for its relief. If the pain is not excessive the application of heat by means of a large poultice, or cold by means of an ice bag, are used to relieve it, or sometimes a few leeches are applied over the painful spot; but the quickest, and probably the most efficient plan, is the hypodermic injection of a little morphia, not necessarily over the seat of the pain, combined, it may be, with one of the other measures. With some physicians, opium in some form is given regularly through the earlier part of the attack. A much-increased rate of breathing and a quickened pulse are two of the usual symptoms of the disease, the breathing—that is the action of the lungs—being increased in greater ratio than the pulse, representing the action of the heart. Should the breathing become excessively rapid, opium, either as Dover's powder or morphia, is given by some, and in the course of from four to six hours lowers the rate of breathing by several respirations a minute, as much as eight or ten in some cases. The pulse rate is also lowered but not in so great a degree. This means much less work for the lungs and heart, and so a certain conservation of energy in the body. Such treatment has not been found to have any injurious effect in stopping the expectoration, which, indeed, may be almost completely absent from the onset of the disease till resolution has taken place; and in addition to lessening the work of the lung, sleep, so often absent or disturbed, is induced. It is not given, or given in much smaller doses, in cases where heart or kidney disease is present, as both, especially the latter class of cases, are very susceptible to the action of this drug.

Turning now to one of the most important points in the treatment of pneumonia—the use of stimulants. Speaking broadly, a straightforward case in a young healthy adult does not require stimulants, and stimulants in such a case often do more harm than good.

The stimulants used are chiefly ammonia—in one or other of its preparations—ether, and alcohol, and of the three the latter is of far and away the greatest use. The quantity given is carefully proportioned according to the indications for its use in each individual case; to a chronic drunkard accustomed to partake daily of a considerable quantity, an amount has to be given far in excess of that required to act as an efficient stimulant in a person unaccustomed to its use.

The pulse and first sound of the heart are perhaps two of the leading indications for the use of stimulants, increasing rapidity and feebleness of the pulse with diminishing strength of the first sound of the heart as heard at its apex, being taken as indications for either one of the special cardiac stimulants or alcohol. To get the maximum of effect from the minimum of alcohol, it is found best to give it frequently, and at regular intervals in doses proportionate to the individual case—one or two tablespoonfuls every four, three, or two hours, as the case may be, always combined if possible with some easily digestible nourishment, such as milk, beef tea, or some of the meat extracts. As has been long taught alcohol has a certain sedative action, and a dose of alcohol at night has a marked effect in lessening or preventing the slighter forms of delirium which are so often associated with an attack of pneumonia. A patient taking alcohol has to be carefully watched so that enough shall be given, but excess avoided.

It is considered that when the pulse becomes slower, the skin moister, the tongue cleaner and moister under its use, alcohol is of benefit; the reverse condition indicates that an excess is being taken.

Neither ether nor ammonia is found to be as efficient, and these are more used for the slighter cases, or else given in conjuntion with alcohol. The form of alcohol used is either brandy or whiskey; some think that when it has to be given in large quantities for a considerable time brandy is better tolerated than whiskey. All forms of spirits used are brought down by means of water to a uniform strength, so that the exact amount of absolute alcohol given is known.

Several of the special cardiac stimulants are largely used in cases where there is increasing cardiac failure; of these the most useful are strychnine, digitalis or its alkaloid digitaline, and strophanthus. Of these the first is the most valuable, and is usually given hypodermically in doses of from 1-150th to 1-50th of a grain, from two to six times in the 24 hours, often combined or alternating with hypodermic injections of digitaline in doses of 1-100th to 1-60th of a grain.

Strophanthus, as the tincture, is given in combination with other drugs, being thought to have greater action than digitalis in strengthening the contractions of the right side of the heart.

The necessity for expectorants varies much in different cases. Some run their course throughout with little or no expectoration and require no expectorants; others with considerable, and perhaps difficult expectoration; and in these latter expectorants are of value. A combination frequently used is one of carbonate of ammonia, squill and senega, as the hospital mist. senegæ co.

practised with great benefit. In cases where with high fever and restlessness there is much engorgement of the right side of the heart, it is found better to bleed several times to the extent of a few ounces than to take a larger quantity at one time.

Amongst the adjuncts to the treatment of pneumonia that have been tried considerably of late mention must be made of the inhalation of oxygen.

Though it undoubtedly prolongs life, eases the breathing, and often restores consciousness, it has no influence on the course of the disease, and though it prolongs, it is seldom that it saves life, at any rate in severe, acute cases.

The oxygen is supplied in long iron cylinders, which contain the gas under a high pressure. At one end of the cylinder is a tap, to which a long rubber tube is attached leading to a large rubber bag; from the bag another tube leads to a face-piece, of which two forms are used, one large like the face-piece of a Clover's ether inhaler, fitting over both nose and mouth, the other a small face-piece to go over the mouth alone, in shape like a large walnut-shell made of ebony, or the gas is given through a piece of tubing placed between the lips; somewhere, either in the tube leading from the bag or on the face-piece, is a tap. The use of the bag is to allow the gas to be given at less pressure than if given direct from the cylinder. The tap in the cylinder being gently opened the bag is filled with gas and the tap closed. The tap near the face-piece, which has been closed during the filling of the bag, is now gently opened, so that the smallest steady stream of gas

Ice Cradle.

Apparatus for Inhalation of Oxygen.

Acting under the belief that the continuance of a high temperature is injurious, some use the ice cradle freely in pneumonia, or apply ice bags to the chest whenever the temperature rises above 103 deg. or 104 deg., till it again falls below the chosen point. The patient being stripped, has placed over his body one or two ordinary iron bed-cradles, to the top of these are suspended small tin buckets full of ice, and covered with flannel to prevent the moisture that condenses on their outsides dropping on the patient, and over the cradle the bedclothes are put. To some the cold is very grateful, others again find it uncomfortable and irksome, and this same difference in apparently similar cases is noticed in a very opposite treatment, namely, hot packing. Most cases have large jacket poultices applied over the affected lung during the time the temperature is raised. After the crisis a gamgee jacket is substituted, as the comfort of a poultice is most appreciated during the febrile stage. In some cases where there is much restlessness and distress, perhaps with slight delirium, the general application of heat to the chest by means of the hot wet pack is used. The time a patient is left in the pack varies according to the result. If found to be soothing and comfortable, he may be left in two or three hours; if after twenty minutes or half-an-hour there is no relief, or increased distress, it is stopped.

The use of depressant drugs, such as aconite and antimony, often of such signal service in sthenic cases, are rarely used, the cases here being chiefly in a class already unable from their surroundings or mode of life to bear any lowering treatment.

Bleeding to the extent of a few ounces is sometimes possible is obtained; the face-piece is held close to the patient's mouth. As the oxygen is inspired the colour of the lips, cheeks, and other parts changes rapidly from purple to red, and the pulse, as a rule, becomes slower.

The oxygen is either given contiuuously or intermittently, in the latter case applying the face-piece only when cyanosis appears. Sometimes the gas in the bag has been given mixed with air, but this is now found unnecessary, as few patients will tolerate a face-piece that fits so tight as to exclude air. Sufficient to dilute the gas is always inspired, and if the gas is given intermittently dilution is not required. The indications for its use are increasing block in the lungs and consequent heart failure, evidenced by the increasing cyanosis and progressive rapidity and feebleness of the pulse.

For the delirium of pneumonia no drug is equal to opium in some form or other, either morphia or Dover's powder being the most generally used. Should opium be contra-indicated, bromide of ammonium or potassium is given. Chloral must be given with caution, and is best combined with a dose of alcohol, or the latter alone will in a slight case ensure sleep.

At, and after the crisis there is always an axious period in a bad case of pneumonia, for the heart, freed from the stimulus of the fever, may tend to fail, and has to be strengthened by heart stimulants such as strychnine, with a judicious quantity of alcohol or other diffusible agent. The poulticing is stopped when the temperature falls, and the patient kept in flannel, or a jacket of gamgee wool next the skin. Food, which during the pyrexial stage has consisted of easily digestible fluid or semi-fluid nourishment, is increased by such solids as the patient is able to take, the rule being to give solid food as soon as it can be taken, and appetite returns fairly rapidly after pneumonia.

Occasionally, instead of resolution taking place in the lung, the pneumonic consolidation persists, though the temperature has fallen and the patient is slowly convalescing; in these cases free counter-irritation by means of iodine is used. Hypodermic injections of pilocarpine have been tried, but not found of much use. The iodides of iron or potash with quinine and other tonics and a liberal diet are given, and the patients are not confined to bed, exercise and fresh air being generally found beneficial.

## Figures and Tables

**Figure f1:**